# True Brachial Artery Aneurysm in Patients with Previous Arterio-Venous Fistula Ligation and Immunosuppressant Therapy for Renal Transplantation: Case Report and Literature Review

**DOI:** 10.3390/healthcare10030470

**Published:** 2022-03-03

**Authors:** Sorin Barac, Andreea Luciana Rata, Alexandra Ioana Popescu, Roxana Ramona Onofrei, Sorin Dan Chiriac

**Affiliations:** 1 Department of Vascular Surgery, Research Centre for Vascular and Endovascular Surgery, “Victor Babes” University of Medicine and Pharmacy Timisoara, 300041 Timisoara, Romania; sorinbarac@gmail.com; 2 Hospital Centre “Saint Nicolas”, “Victor Babes” University of Medicine and Pharmacy Timisoara, 57400 Sarrebourg, France; alexandra_popescu2007@yahoo.com; 3 Department of Rehabilitation, Physical Medicine and Rheumatology, “Victor Babes” University of Medicine and Pharmacy Timisoara, Research Center for Assessment of Human Motion, Functionality and Disability, 300041 Timisoara, Romania; onofrei.roxana@umft.ro; 4 3rd Surgery Department, “Victor Babes” University of Medicine and Pharmacy Timisoara, 300041 Timisoara, Romania; chiriac.sorin@umft.ro

**Keywords:** brachial artery aneurysm, immunosuppression, renal transplantation, arterial interposition

## Abstract

Background/Objective: Brachial artery aneurysm (BAA) is a serious complication in patients with previous arterio-venous fistula (AVF), renal transplantation (RT), and immunosuppressive regimens. Until now, there has been no standard of care for these patients, especially for patients undergoing chronic dialysis and immunosuppressive treatment. The aim of this study was to investigate data from the literature regarding these patients and to suggest recommendations for the best approach to their treatment. Methods: A review of the literature was performed by searching the PubMed database in the English language. The review was accompanied by two case reports. A total of 24 articles with different variables—demographics, renal transplantation, aneurysm size, and type of surgery—were subjected to the review. In addition, two cases are reported. Conclusion: This review suggests that the best treatment for these patients is open surgery, with aneurysmectomy and graft interposition. Results: All patients had RT. The age of patients ranged from 26–77 yo, with a male predominance. The majority had an AVF ligated after RT. The main clinical symptoms were pain, swelling, and pulsatile mass (66%). All patients, except one, were treated through open surgery. The first option for treatment was reversed saphenous vein graft interposition (36%), followed by ePTFE graft (16%).

## 1. Introduction

Upper-extremity peripheral aneurysms are rare and account for less than 1% of all peripheral artery aneurysms. The brachial artery is involved around 0.5% of cases, with 0.17% being true aneurysms [1].

Brachial artery aneurysm (BAA) in patients with previously ligated arterio-venous fistula (AVF) for hemodialysis and renal transplantation (RT) is rare and few cases are presented in the literature [2].

The number of patients that require RT is increasing worldwide. In 2017, there were 90,306 RTs worldwide, both from live and deceased donors [3]. All included patients that required kidney transplantation and who had AVF access for hemodialysis. In these patients, high flow due to AVF and an immunosuppressant regimen in the case of transplantation are risk factors for brachial artery dilatation [4,5].

This complication is extremely challenging, mostly due to the general profile of the patient, especially for patients on chronic dialysis and immunosuppressive regimens.

BAA is associated with rare complications, such as distal limb ischemia, aneurysm rupture, nerve compression, and upper limb swelling [6].

In this study, we present a literature review and two case reports of BAA, with the aim of evaluating the treatment modalities used for these patients.

## 2. Materials and Methods

Two cases of giant BAA in patients with chronic kidney disease (CKD) and RT are presented below. The patients treated gave their informed consent for the use of their personal data. The article has the approval of the Ethics Committee of the “Pius Brânzeu” Clinical County Emergency Hospital no. 188/4 May 2020. Both patients gave their consent for publication.

A literature review was performed by searching the PubMed database. The terms used for the search were brachial artery aneurysm, AVF ligation, and RT. The search was limited to any reported cases of brachial artery aneurysm formation in patients with RT and previous AVF ligation. The reports were selected according to the PRISMA guidelines. In the end, we selected 26 articles that matched all our keywords.

**Figure 1:**
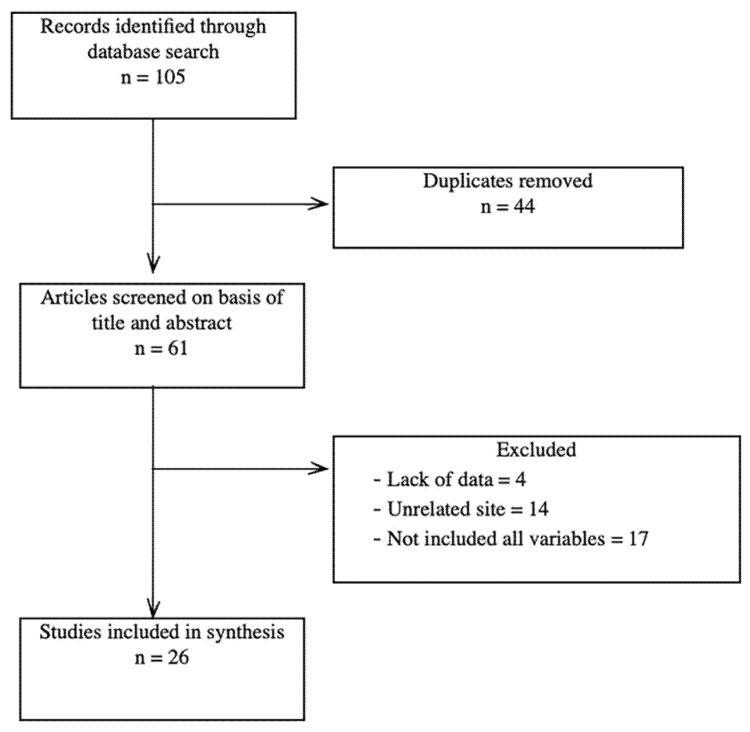
PRISMA flow diagram.

Figure 1 shows a PRISMA flow diagram for this review. We conducted PubMed and Medline database searches in the English language. The keywords we used were “brachial artery aneurysm”, “arteriovenous fistula”, “immunosuppressive treatment” and “renal transplantation”. After the removal of duplicates we screened 61 articles on basis of their titles and abstracts. We excluded 35 articles due to a lack of data (absence of AVF age, type of conduit used for vascular reconstruction, exact location of the aneurysm), an unrelated site of aneurysm formation, or due to the fact that they did not include all the variables (Figure 1).

The variables included in our search were related to the age of the AVF at the time of ligation, the duration from the ligation until aneurysm development, the type of clinical presentation, treatment choice, and type of immunosuppressive treatment.

## 3. Results

### 3.1. Case Reports

**Figure 2:**
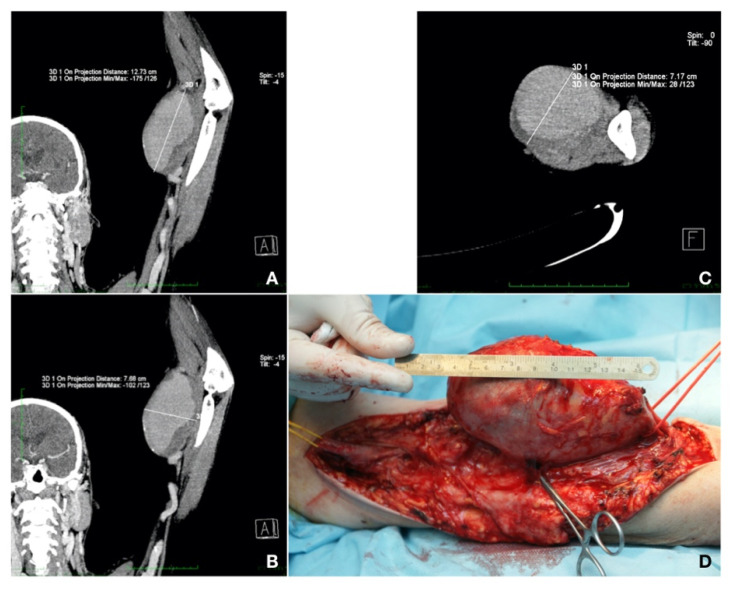
(**A**,**B**) Longitudinal maximum intensity projection (MIP) section showing the aneurysm. (**C**) Transversal section. (**D**) Intraoperative image showing the dimensions of the aneurysm and proximal (yellow loop) and distal (red loop) ends of the vessels to be anastomosed with an inverted saphenous graft (personal collection).

Case 1. A 59-year-old patient presented with a left brachial mass that had developed during the past 3 years. The patient also had type 2 diabetes mellitus, treated with diet, and dyslipidemia, treated with a statin. The mass was approximately 10 cm in size, pulsatile, but without any particular symptoms. The patient was known for chronic glomerulonephritis and chronic renal disease stage 5 KDIGO for 5 years. Three years previously, he had undergone live donor renal transplantation and, since then, he had been on immunosuppressive and steroid agents (fujimycin 0.5 mg, mycophenolic acid 2 × 180 mg, and prednisone 5 mg daily). After the RT, the AVF on the left side was closed. The patient was currently undergoing chronic RT rejection and hemodialysis three times per week. A CT-Angio of the left arm showed a brachial artery aneurysm of 12.73/7.68 cm, partially thrombosed. Aneurysm resection and vascular reconstruction with spatulated end-to-end anastomosis were performed [7]. The patient received preoperatively an adjusted dose of Ceftriaxone^®^ (Antibiotice S.A., Iasi, Romania) at 1 g/dosage for infection prevention. An aneurysmectomy and reversed saphenous vein graft interposition, end-to-end anastomosis with 6.0 Prolene^®^ (BBraun, Hessen, Germany) continuous sutures were performed under general anesthesia. The postoperative evolution was without complications (embolic, neurological, or surgical site bleeding or infection) and the patient was discharged 10 days after surgery (Figure 2).

**Figure 3:**
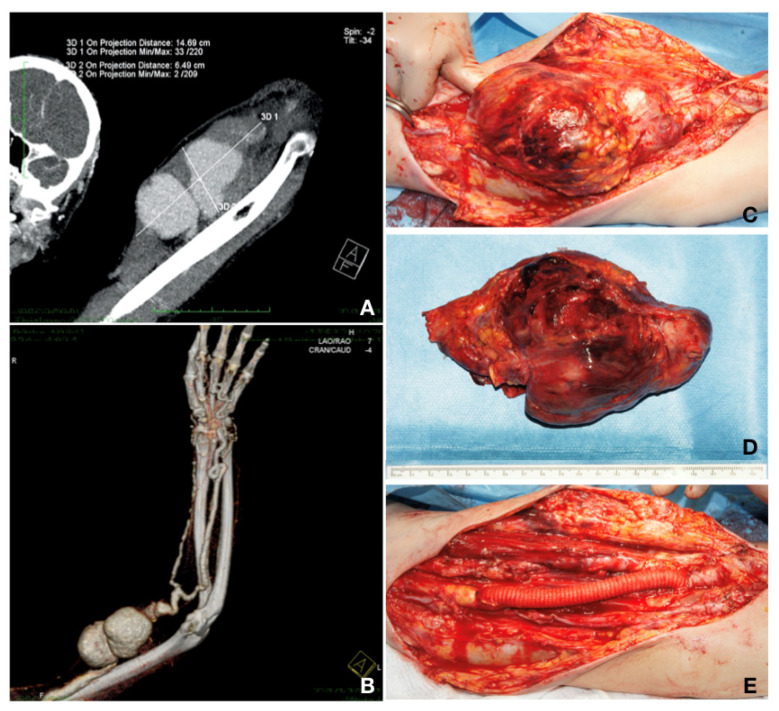
(**A**,**B**) MIP thin collection and volume rendering technique (VRT) CT-Angio showing the 2D dimensions of the giant aneurysm. On the VRT image, one can observe the bad quality of the radial artery and the calcified aspect of both run-off vessels distal to the aneurysm. (**C**) Intraoperative image with the aneurysm dissection. (**D**) Resected aneurysm with mural thrombosis. (**E**) Vascular reconstruction with 10 mm Dacron graft interposition and spatulated, end-to-end anastomosis with Prolene 6.0 continuous sutures (personal collection).

Case 2. A 42-year-old patient presented with a pulsatile mass in the upper left arm, with a skin erosion and the loss of the grip strength, also known to have chronic renal disease stage 5 KDIGO and Alport syndrome, and having undergone a live-donor RT 8 years prior. The patient had palpable radial and ulnar pulses. He had, on the same arm, a radio-cephalic AVF, closed 6 years before with radial artery reconstruction and cephalic vein thrombosis. He was on immunosuppressive and steroid agents (mycophenolic acid 2 × 180 mg and prednisone 5 mg daily). He underwent a CT-Angio investigation that showed a brachial artery aneurysm of 14.69/6.49 cm. The patient did not require hemodialysis after the investigation because the level of creatinine was constant at 4 mg/dL at 48 and 72 h after the investigation, respectively. Furthermore, for this patient, aneurysm resection and vascular reconstruction were performed. The patient received preoperatively an adjusted dose of Vancomycin^®^ (Fresenius Kabi, Brasov, Romania) at 1 g/dosage for infection prevention. Aneurysm resection and reconstruction with a collagen-impregnated woven nylon graft (Dacron^®^; Vascutek, Terumo) of 10 mm in diameter and end-to-end anastomosis with 6.0 Prolene^®^ (BBraun, Hessen, Germany) continuous sutures were performed under general anesthesia. This vascular graft was chosen due to the arterial diameter (more than 10 mm), arterial wall thickness (approximately 2 mm and anfractuous structure), and because the saphenous vein of the patient was not fit for the procedure (1.5 mm in diameter, as previously measured by ultrasound). The postoperative evolution was without complications on the surgical site (embolic, neurological, bleeding, or infection at the surgical site). A week after the vascular reconstruction, the patient developed constrictive pericarditis and needed a pericardial window. Two months after surgery, the patient developed a chronic rejection of the transplanted kidney and required nephrectomy. The status of the patient deteriorated rapidly, and he died 3 months after reconstruction (Figure 3).

Both patients underwent preventive management of contrast-induced nephropathy according to the KDIGO Clinical Practice Guidelines for acute kidney injury [8,9].

Both patients were on a full association of antihypertensive drugs (calcium blockers, diuretics, angiotensin-converting enzyme inhibitors, clonidine, and β-blockers).

The incision was made along the aneurysm projection to the skin. A drainage was left in place and it was removed after 24 h. The skin was closed with skin staples.

After arterial reconstruction, the patients were treated with antiplatelets, aspirin (case 1) and clopidogrel (case 2) at 75 mg daily, respectively.

In both patients, the one-month follow-up showed no pain or neurological deficits (numbness, paresthesia, or nerve compression symptoms), and the surgical site incision healed, and the distal radial and ulnar pulses were palpable.

### 3.2. Evidence from the Literature

BAA is defined as an arterial enlargement of more than 50% of the normal diameter. For the brachial artery, the normal diameter ranges from 3.5 to 4.3 mm in women and 4.1 to 4.8 mm in men, respectively [10].

Fifty-six BAA cases were reported in the literature [2,4,5,11,12,13,14,15,16,17,18,19,20,21,22,23,24,25,26,27,28,29,30,31,32,33,34]. The mean age of patients was 50.94 yo (ranging between 26 and 77 yo). The ratio of men to women was 7:1, i.e., with 43 males (84.3%) and 6 females (11.76%). All patients had an RT and an AVF for hemodialysis. Regarding the type of AVF, this aspect is detailed in Table 1.

The age of AVF was not mentioned in all cases but, when mentioned, it ranged from 12 to 354 months (with a mean age of 99.13 months). Of all AVFs, seven were not ligated, in 25 cases the duration after the ligation was not mentioned, and the mean duration of the ligation was 93 months, ranging from 6 to 252 months. The common presentation was with pain, swelling, and a pulsatile mass (66.66%—34 patients), followed by nerve compression (seven patients—13.72%), and ischemia (four patients—7.84%). It was also observed that most of the aneurysms were in the left arm, probably because the AVF was made on the non-dominant arm.

The surgical conduit was as follows: reversed saphenous vein—20 patients (35.71%), ePTFE graft—eight patients (15.6%), end-to-end reconstruction—six patients (11.76%), reversed cephalic/basilic vein—eight patients (15.68%), other interventions (no reconstruction, endovascular procedure, allograft interposition, reducing diameter, femoral artery transposition—14 patients (25%).

An immunosuppressive regimen was present in all patients, but not specified in 38 cases; other immunosuppressive regimens included methylprednisolone, azathioprine, tacrolimus, and mycophenolate mofetil.

In this review, a male preponderance for BAA was found, but the reason for this is still unknown [35].

## 4. Discussion

The first physician who described a proximal dilatation after the ligation of an AVF fistula was William Hunter in 1757 [36].

In the studied cases, there were 18 patients with reversed saphenous graft interposition (36%), eight patients with ePTFE (16%), six with end-end reconstruction (12%), four with inversed basilic veins (8%), three with inverted cephalic veins (6%), one with an allograft reconstruction, one with a cadaveric brachial artery, one with a devalvulated saphenous vein, and one with femoral artery transposition.

The first case was fit for reconstruction with a saphenous vein but, in the second case, a synthetic graft was preferred because of the diameter of the artery (Dacron vs. ePTFE was chosen for the graft due to its flexibility and because we thought it was easier to suture it on a fragile arterial wall) and because the patient did not have a suitably large saphenous vein, as seen on the ultrasound examination.

Another treatment method is the endovascular approach, with the placement of a stent graft, but the risk of contrast-induced nephropathy in these patients is very high. The placement of a stent graft is a challenging technique for the treatment of a BAA. First of all, it is associated with a high risk of embolization and a superficial position of the brachial artery that can be easily compressed by extrinsic factors and arm movements (pronation, supination, flexion, extension) and stent damage. There is also discussion of the remnant arterial aneurysm that can compress the nervous structures of the arm, especially in case of a large aneurysm [10]. Further studies need to be conducted to find the right place for endovascular treatment in these patients, also taking into the account the high associated surgical risk.

There are several known mechanisms that can be involved in arterial degeneration: the arterial wall stress, the long-term use of immunosuppressive agents, and the chronic inflammation caused by various factors [37].

The stress on the arterial wall is mediated by the production of free oxygen radicals. They combine with NO, lead to the production of peroxide nitrates, and mediate vessel dilatation. Consequently, the arterial wall exceeds its adaptive capacity, thus leading to an inflammatory response which promotes smooth muscle cell differentiation, proliferation, and migration, and subsequent wall thickness [38].

Chung et al. demonstrated that the degradation of elastic fibees extends to all layers of the arterial wall, and also stated that a longer duration of dialysis leads to a higher amount of calcium and phosphate deposition [39].

The long-term use of immunosuppressive agents leads to vascular remodeling on each of the three layers of the artery. This phenomenon is mediated by an inflammatory process activated by cytokines [40,41].

There are also other mechanisms by which the arterial wall is subject to chronic inflammation: first of all, through hemodialysis itself, followed by diabetes, hyperlipidemia, arterial hypertension, infection, and repetitive trauma [37].

This type of complication of the vascular access for hemodialysis in RT patients is a challenging one due to the patient profile, especially in relation to CKD and the immunosuppressive regimen. Considering these issues, a vein conduit is the first choice, followed by an ePTFE graft. In our case we chose Dacron^®^ (Vascutek, Terumo) without spiral support because we did not pass the elbow joint.

## 5. Conclusions

The pathogenesis involved in the formation of BAA in patients with RT, immunosuppressive regimens, and AVF ligation consists of an association of arterial wall stress, the action of immunosuppression (through cytokine activation and vascular wall remodeling) and chronic inflammation.

In patients with a giant BAA, RT, and an immunosuppressive regimen, the gold standard for aneurysm repair remains open surgery with aneurysm excision and arterial reconstruction. The role of endovascular therapy is yet to be determined.

We also recommend periodic ultrasound follow-up on patients with a previous ligation of an AVF and immunosuppressive treatment in order to catch this complication in its early stages.

## Figures and Tables

**Table 1:** Distribution of patients with respect to AVF type.

Type of Fistula	No of Patients	%
Brachio-basilic	1	1.78
Brachio-cephalic	24	42.85
Radio-cephalic	20	35.71
Brachio-/Radio-cephalic	1	1.78
Not mentioned	10	17.85

## Data Availability

Not applicable.
